# An acquired high-risk chromosome instability phenotype in multiple myeloma: Jumping 1q Syndrome

**DOI:** 10.1038/s41408-019-0226-4

**Published:** 2019-08-09

**Authors:** Jeffrey R. Sawyer, Erming Tian, Brian A. Walker, Christopher Wardell, Janet L. Lukacs, Gael Sammartino, Clyde Bailey, Carolina D. Schinke, Sharmilan Thanendrarajan, Faith E. Davies, Gareth J. Morgan, Bart Barlogie, Maurizio Zangari, Frits van Rhee

**Affiliations:** 1 Department of Pathology, University of Arkansas for Medical Sciences, Little Rock, AR USA; 2 Myeloma Center, University of Arkansas for Medical Sciences, Little Rock, AR USA; 3 Department of Hematology and Medical Oncology, Tisch Cancer Institute, Icahn School of Medicine at Mount Sinai, New York, NY USA

**Keywords:** Genetics research, Cancer epigenetics

## Abstract

Patients with multiple myeloma (MM) accumulate adverse copy number aberrations (CNAs), gains of 1q21, and 17p deletions during disease progression. A subset of these patients develops heightened 1q12 pericentromeric instability and jumping translocations of 1q12 (JT1q12), evidenced by increased copy CNAs of 1q21 and losses in receptor chromosomes (RC). To understand the progression of these aberrations we analyzed metaphase cells of 50 patients with ≥4 CNAs of 1q21 by G-banding, locus specific FISH, and spectral karyotyping. In eight patients with ≥5 CNAs of 1q21 we identified a chromosome instability phenotype similar to that found in ICF syndrome (immunodeficiency, centromeric instability, and facial anomalies). Strikingly, the acquired instability phenotype identified in these patients demonstrates the same transient structural aberrations of 1q12 as those found in ICF syndrome, suggesting similar underlying pathological mechanisms. Four types of clonal aberrations characterize this phenotype including JT1q12s, RC deletions, 1q12-21 breakage-fusion-bridge cycle amplifications, and RC insertions. In addition, recurring transient aberrations include 1q12 decondensation and breakage, triradials, and 1q micronuclei. The acquired self-propagating mobile property of 1q12 satellite DNA drives the continuous regeneration of 1q12 duplication/deletion events. For patients demonstrating this instability phenotype, we propose the term “Jumping 1q Syndrome.”

## Introduction

Chromosome instability (CIN) is defined as an increased rate and ongoing acquisition and accumulation of copy number aberrations (CNAs), and not simply the existence (state) of structurally and numerically abnormal aneuploid clones^1,2^. The most common CIN phenotypes are either whole-chromosome gains or losses or unbalanced structural aberrations leading to segmental focal amplifications and deletions. CIN phenotypes are thought to contribute to tumor progression and drug resistance through the amplification of oncogenes and/or loss of tumor suppressor genes and will require novel therapeutic interventions^3,4^. In multiple myeloma (MM), numerical and structural chromosome aberrations are most commonly identified by interphase fluorescence in situ hybridization (iFISH)^5^, which provides guidance for patient stratification and therapeutic intervention^6^. The International Myeloma Working Group (IMWG) has designated a group of adverse IgH translocations including t(4;14), t(14;16), and t(14;20), and adverse CNAs including deletions of 17p and gains of 1q21^7,8^. The accumulation of different combinations of these adverse iFISH lesions during tumor progression has been reported to be a factor contributing to high-risk disease and eventual relapse^9^.

In MM, whole-chromosome CNAs are reflected in either hyperdiploid or nonhyperdiploid clones, which are not indicative of CIN, since both of these aneuploidy clones generally remain stable during the course of the disease^5^. CIN phenotypes also include arm length and smaller segmental CNAs involving focal amplifications and deletions that are best identified by single-nucleotide polymorphism (SNP) microarrays. SNPs microarray analysis has shown that segmental CNAs are frequent in MM, and that 1q21 is not only one of the most frequent CNAs in this disease^10–14^ but in all cancers^15^. Microarray studies have shown that in a typical cancer 25% of the genome is affected by arm-level CNAs, while only 10% by focal CNAs^16^. Importantly, in MM the gains of the 1q21 region occur most commonly as arm-level aberrations, but also occur as focal amplifications, both of which have been shown to result from 1q12 instability^17–21^. The 1q21 region is known to contain a number of putative oncogenes and genes that may show the simultaneous amplification and/or deregulated expression, including MCL1, IL6R, BCL9, CKS1B, ANP32E, ILF2, and ADAR1^22–24^. Gains of a single copy of 1q (CN 1q21 of 3) are found in about 30% of newly diagnosed patients, while in highly proliferative disease or relapsed patients the accumulation of additional 1q21 CNAs result in as many as 70% of patients having four or more 1q21 aberrations^25^. Increasing copy numbers of 1q21 are associated with a worse prognosis^25^. The accumulation of CNAs of 1q21 evidenced by the progressive gain of 1q21 from 3 to 4 and 5 copies is significant because it serves as a marker for chromosomal instability, including whole-arm gains and losses, breakage-fusion-bridge (BFB) cycle gene amplification, and dispersed insertions. Recent comprehensive genomic analysis indicates that the nonrandom accumulation of genetic hits occurs on top of the primary events in myeloma^26^, and that high-risk subgroups can be defined by either biallelic deletion of *TP53* or amp 1q21 (≥4 copies) on the background of ISS III^27^. Unfortunately, the underlying mechanisms for the genomic CNAs, such as del(17p) and gain of 1q21, are still poorly understood.

We have previously reported increasing CNAs of 1q21 occurring as jumping translocations of 1q12 (JT1q12) and that these translocations are the cause of unbalanced aberrations in the receptor chromosomes (RCs)^17–21^. A subset of these patients with CNAs ≥5 1q21 has shown an increased level of site-specific instability in 1q12 satellite DNA. Importantly, the 1q12 satellite DNA region in these patients acquires a self-replicating mobile property that is demonstrated by the ability to duplicate the 1q12 region and to “jump” to different nonhomologous chromosomes. This 1q12 CIN is continually regenerated in subsequent cell cycles, resulting in both novel transient and clonal 1q12 aberrations. This intrachromosomal CIN strongly suggests a mechanistic link to the CIN phenotypes found in the autosomal recessive (AR) CIN syndromes, most notably ICF syndrome (immunodeficiency, centromeric instability, and facial anomalies)^28^. AR CIN syndromes include Fanconi anemia, Bloom syndrome, and ataxia telangiectasia, all of which have distinct CIN phenotypes^29^. These disorders show intrachromosomal aberrations including breaks, gaps, chromatid exchanges, and multiradial chromosomes, with both Fanconi and ICF syndromes showing unbalanced gains of 1q as part of their cytogenetic phenotypes. In particular, ICF patients show hallmark aberrations including site-specific 1q12 pericentromeric instability involving 1q12 decondensation, 1q12 triradials, multibranched chromosomes 1q, isochromosomes 1q (iso 1q), unbalanced translocations with 16q, and 1q micronuclei in blood cultures^29,30^. Strikingly, myeloma patients with highly proliferative disease and high CNAs of 1q21 share these same transient 1q12 chromosome aberrations, but also display a spectrum of clonal 1q21 CNAs. A defining feature of this CIN phenotype in MM is the continuous regeneration of unstable aberrations of 1q12 that results in an ongoing accumulation of both arm-level and focal CNAs^17–21^. The distinction between the 1q12 instability in ICF syndrome and that seen in MM is that in ICF the instability is transient, found only in blood, while in MM the 1q12 aberrations become clonal and continue to drive subclonal copy number heterogeneity.

To investigate the structural relationships of 1q12 aberrations to the accumulation of 1q21 CNAs during disease progression, we undertook a metaphase FISH study of 50 patients with ≥4 copies of 1q by G-banding. This analysis revealed a subset of patients with high-risk IgH aberrations and/or 17p deletions and ≥5 copies of 1q21. We now recognize and report the cytogenetic features of a distinct acquired 1q12 CIN phenotype. These patients show profound chromosome 1q12 instability including the major clonal features of JT1q12 gains, losses of RC arms including 17p, amplification of the 1q12-23 region by BFB cycles, and dispersed insertions of nonhomologous chromosome segments. Transient features of this phenotype include the decondensation and breakage of 1q12, acentric lagging chromosomes 1q, 1q21 micronuclei, and localized shattering of 1q distal to 1q12. In addition, the identification of different copy numbers or the switching of copy numbers can occur in different subclones. For this constellation of 1q12 cytogenetic findings, we propose the term “Jumping 1q Syndrome.” To provide guidance for patient stratification and therapeutic intervention this phenotype can be identified by IMWG iFISH panels by the presence of ≥5 CNAs of 1q21 concurrent with the detection of at least one other adverse iFISH lesion.

## Patients and methods

The Institutional Review Board of the University of Arkansas for Medical Sciences approved the research studies, and all subjects provided written informed consent approving the use of their samples for research purposes. Metaphase chromosomes were prepared from bone marrow, fine needle aspirate, and pleural effusion specimens, and processed for G-band analysis as previously described^17^.

Patient sample selection was based on the suspected presence of at least two additional copies of 1q (≥4); only patients with ≥4 copies of 1q21 confirmed by metaphase FISH were included in this study. Specimens with unresolved aberrations by FISH were further scrutinized by spectral karyotyping (SKY).

### FISH and SKY methods

Probes used to demark the pericentromeric regions of chromosomes including sat III (1q12), sat II (D16Z3)(16q11), and alpha sat (9q12) were used according to the manufacture's protocol (Vysis, Downers Grove, IL). Two probe sets for the detection of IgH translocations included IGH/FGFR3 dual color, dual fusion probe set and the IGH dual color, break-apart rearrangement probe (Vysis). FISH probes for MCL1 (1q21), CKS1B (1q21), TP53 (17p13), and ERBB2 (17q12) were prepared as previously described^20^. The SKY probe mixture and hybridization reagents were prepared by Applied Spectral Imaging (Carlsbad, CA) and procedure performed as previously described^20^. Image acquisition for FISH and SKY was performed using an SD200 Spectracube (Applied Spectral Imaging, Inc.) mounted on a Zeiss Axioplan II microscope (Gottingen, Germany). DAPI images were captured, and then inverted and enhanced by SKY View software to produce G-band-like patterns on the chromosomes^20^. Original magnifications of all G-band and FISH images were ×1000. Original magnification for SKY images was ×630.

## Results

Metaphase FISH results and clinical characteristics for all 50 patients are provided in Supplemental Table 1, and complete G-band karyotype designations^31^ are provided in Supplemental Table 2. FISH analysis showed 27 patients with four copies of 1q21 (nos 3, 9, 10, 12, 13, 15, 16, 17, 19, 24, 25, 26, 27, 28, 29, 33, 34, 35, 36, 39, 40, 41, 43, 44, 45, 48, and 50), while 23 patients had ≥5 copies (nos 1, 2, 4, 5, 6, 7, 8, 11, 14, 18, 20, 21, 22, 23, 30, 31, 32, 37, 38, 42, 46, 47, and 49). Patients with the IgH translocations t(4;14), t(14;16), and t(14;20) were grouped together and designated as adverse IgH lesions. Seventeen patients showed adverse IgH lesions (nos 2, 4, 7, 16, 17, 18, 19, 20, 21, 25, 27, 39, 43, 45, 46, 47, and 49). Six patients (nos 6, 8, 9, 24, 41, and 50) showed t(11;14), eight (nos 1, 11, 13, 32, 33, 37, 38, and 44) showed del 14q32 or -14, and one (14) with t(8;14). Eighteen patients (nos 3, 5, 10, 12, 15, 22, 23, 26, 28, 29, 30, 31, 34, 35, 36, 40, 42, and 48) were normal for IgH FISH. FISH identified 22 patients with deletion 17p (nos 3, 4, 5, 8, 9, 14, 16, 19, 22, 24, 29, 30, 34, 36, 37, 39, 40, 42, 43, 45, 49, and 50) and 28 normal. Five showed RC deletions of 17p by JT1q12 (nos 3, 30, 42, 43, and 49) (Supplemental Table 1).

### Jumping translocations of 1q12 and concomitant deletions in RCs

Among the total group of 50 patients, 36 with JT1q12 showed a concomitant deletion in an RC, while 14 showed no deletion in an RC (Supplemental Table 1). Recurring arm-length deletions in five or more patients were identified in chromosome arms 1p, 19q, 6q, 16q, and 17p (Supplemental Table 1). Among the 23 patients with ≥5 CNAs of 1q21 recurring arm-length losses were found in 1p (nos 2, 4, 6, and 7), 6q (nos 1, 14, and 46), 17p (nos 30, 42, and 49), 16q (nos 20, 32, 37, and 38), 19q (nos 22 and 31), and six with no deletion (Table 1).

**Table 1:** Ploidy levels and metaphase FISH results for 23 patients with ≥5 copies for 1q21

	Ploidy	IgH trans	1q12 CN	1q21 CN	17p CN	1q12 trans to RC	1q12 deletion in RC
1	NHRD	del(14q32)	6	6	2	der(1;6)	del 6q
2	NHRD	t(4;14)	5–10	5–6	2	der(1;8)	del 1p
4	NHRD	t(4;14)	4–6	5–7	1	iso 1q	del 1p
5	HRD	Normal	5	5	1	der(1;12)	None
6	NHRD	t(11;14)	5–6	5–6	2	iso 1q	del 1p
7	NHRD	t(4;14)	5–9	5–9	2	iso 1q	del 1p
8	NHRD	t(11;14)	6–8	6–8	1	iso 1q	del 1p
11	NHRD	del(14)	5–7	5–7	2	quad 1q, trip 1q21	None
14	HRD	t(8;14)	5–6	5–6	1	der(1;19)	del 6q
18	NHRD	t(4;14)	5–7	5–7	2	dup 1q, der(1;7)dup 2	None
20	NHRD	t(4;14)	6	6	2	der(1;16)	del 16q
21	NHRD	t(4;14)	5	5	2	der(1;7)	None
22	HRD	Normal	5–9	5–9	1	der(1;19)	del 19q
23	HRD	Normal	5	5	2	dup 1q, der(1;13)	None
30	NHRD	Normal	5–6	5–6	1	der(1;9p)der(1;17)	del 17p
31	NHRD	Normal	7	7	2	der(1;19)	del 19q
32	NHRD	del(14q32)	6	6	2	der(16), der(1;22)	del 16q
37	NHRD	del(14q32)	5	5	1	der(1;16),der(1;20)	del 16q
38	NHRD	del(14q32)	5	5	2	der(1;16)	del 16q
42	HRD	Normal	5	5	1	der(1;17)	del 17p
46	NHRD	t(4;14)	5	5	2	der(1;6), dup 1q	del 6q
47	HRD	t(4;14)	5	5	3	der(1;2), der(19), der(22)	None
49	NHRD	t(4;14)	5	5	1	der(1;17)	del 17p

IgH translocations, CNAs for 1q12, 1q21, 17p, and receptor chromosome gains and losses related to 1q12. Chromosome ploidy levels defined by hyperdiploid (HRD) equaling 47–75 chromosomes and nonhyperdiploid (NHRD) equaling 46 and/or >75 chromosomes

**Fig. 1:**
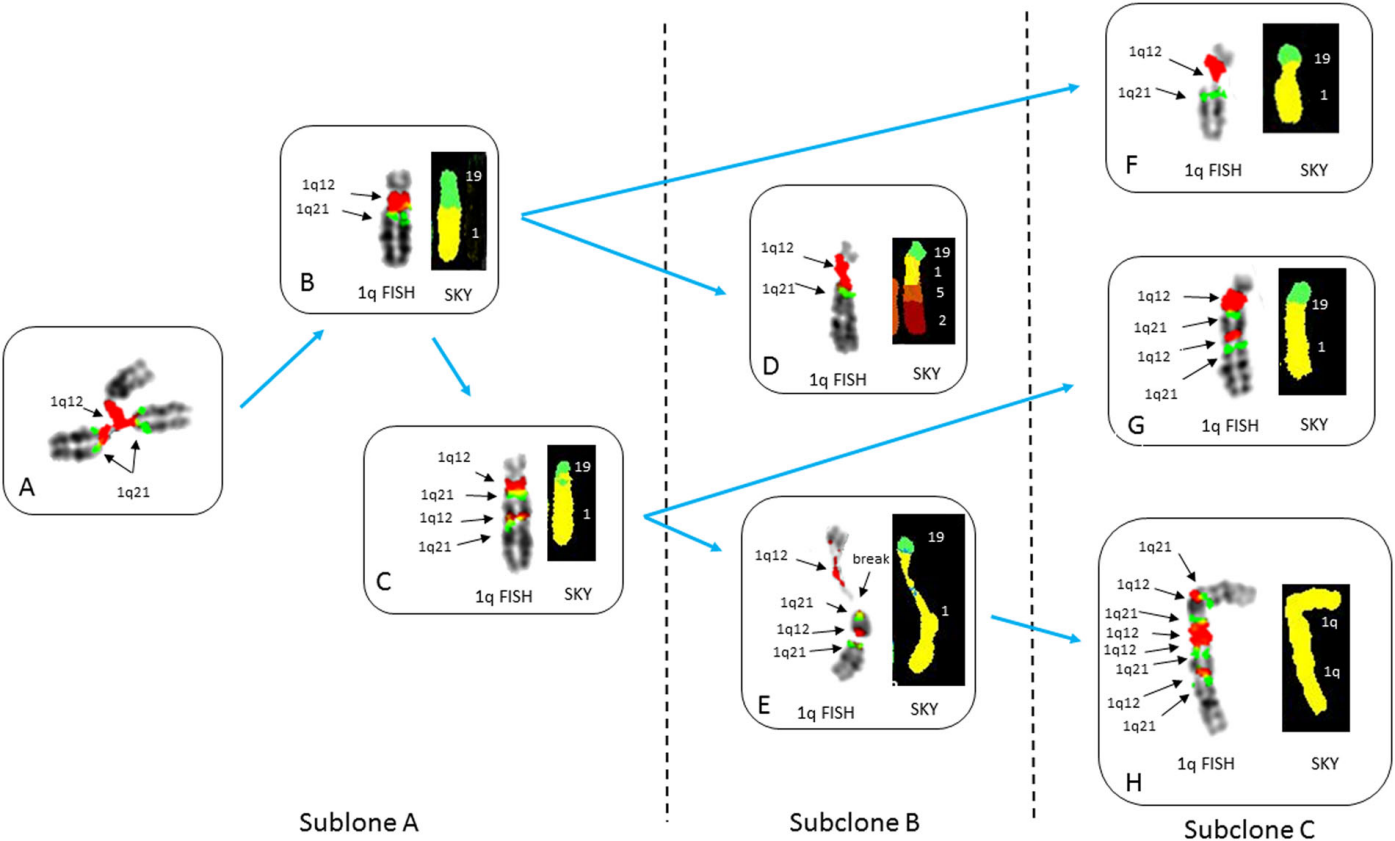
Partial karyotypes from patient no 22 demonstrating subclonal heterogeneity and the ongoing regeneration 1q21 CNAs. FISH probes 1q12(red) and 1q21 (green) and SKY classified colors for chromosomes 1 (yellow), 2 (crimson), 5 (orange), and 19 (green) (**a–h**). Blue arrows depict evolving lineage of the 1q12 aberrations appearing in the different subclones. Subclone A demonstrates transient triradial chromosome 1q12 (**a**), which regenerates extra copies of 1q21 (arrows). Note branching of 1q12 pericentromeric heterochromatin (arrow) leading to the duplicate 1q arm displaying the extra copy of the 1q21. **b** A JT1q12 to RC19 results in a der(19) with gain of 1q and a concomitant whole-arm deletion of 19q. **c** A reduplication of the der(19) shows an additional direct duplication of the 1q12-q21. A total of six copies of 1q21 were identified in this subclone (normal chromosome 1 not shown). In subclone B (**d**, **e**), the evolution of der(19)s is demonstrated by a complex multichromosome rearrangement in one of the der(19)s (**d**). SKY identified segments of chromosomes 5 and 2 translocated distal to 1q21 (**d**). The other der(19) in this subclone demonstrates the transient decondensation and breakage in the 1q12 pericentromeric heterochromatin (**e**). This subclone shows five copies of 1q21 (normal chromosomes 1 not shown). The further progression of 1q12 aberrations in subclone B is evidenced by the new aberrations found in subclone C, which shows the decondensation and breakage in 1q12 (**f**), and the generation of a novel iso (1q) with four copies each of 1q12 and 1q21 (**h**). This subclone shows a total of nine copies of 1q21 (normal chromosomes 1 not shown)

We identified a subset of eight patients with ≥5 of 1q21, arm-length deletions (nos 2, 4, 7, 14, 22, 30, 42, and 49), and at least one other high-risk iFISH lesion (Table 1) that showed ongoing instability of 1q12 in their metaphase cells. These patients showed continuous regeneration of novel 1q12 and 1q21 CNAs, with concomitant losses in RCs. For example, patient no 22 showed regenerating JT1q12 instability with a range of CNAs for 1q21 from 5 to 9 and multiple sequential aberrations to chromosomes 19 (Fig. 1a–h). The driver of 1q21 CNAs in this patient is evidenced by the presence of the hallmark triradial of 1q12, which propagates the extra copy to 1q21 (Fig. 1a). In this patient, the JT1q12 jumped to RC19 deleting the 19q (Fig. 1b), which subsequently reduplicates and shows two copies of 1q21 (Fig. 1c). In a second more complex subclone a multichromosome rearrangement distal to 1q21 is identified by SKY (Fig. 1d), and continued decondensation and breakage of 1q12 is seen (Fig. 1e). In a third subclone (Fig. 1f–h), an isochromosome 1q is identified containing four copies of 1q21 (Fig. 1h). The continuous 1q12 instability on the RCs 19q resulted in subclones containing different copy numbers of 1q21, in different configurations (Fig. 1a–h).

**Fig. 2:**
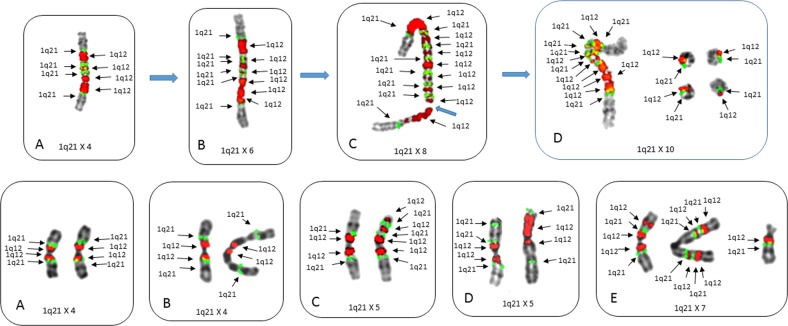
Metaphase cells from patients demonstrating different types of progression events involving isochromosomes 1q. Top patient no 7 (**a**–**d**) showing progressive gains of both 1q12 and 1q21 resulting from BFB cycle amplifications of the 1q12–1q21 region. The 1q12 breakpoints generate gains of 1q21 in extended ladder-like pattern. FISH probes 1q12(red) and 1q21 (green). Normal chromosomes 1 from these cells (**a**–**d**) not shown. **a** An isochromosome 1q showing an alternating pattern of 1q12 and 1q21 with four copies of each (arrows). **b** Increased copy number amplification of both 1q21 and 1q12 resulting in six copies of both 1q12 and 1q21 in this chromosome. **c** The iso(1q) in this cell shows the characteristic alternating copies of 1q12 and 1q21 and extended ladder-like structure seen in BFB cycles demonstrating eight copies of both 1q12 and 1q21. **d** A cell with a total of ten copies of both 1q12 and 1q21 with six copies on the iso(1q) and four small extra-chromosomal acentric rings each with single copies of 1q12 and 1q21. Bottom Partial karyotypes from patient no 4 demonstrating two isochromosome 1qs with intra-arm heterogeneity of both CNAs and loci position of 1q12 and 1q21. FISH probes 1q12 (red) and 1q21 (green). **a** This cell shows the expected copy number and loci position for two “normal” isochromosomes 1q with four copies of 1q12 and 1q21. **b** The iso(1q) on left shows normal copy number and positions for 1q12 and 1q21, while the iso(1q) on the right shows the 1q21 locus has inverted distally in both arms. **c** The iso(1q) on left shows the normal copy number and positions for 1q12 and 1q21; however, the iso(1q) on right shows four copies of 1q12 and three copies 1q21 (arrows) with unequal CNAs between the arms. **d** Both iso(1q)s show intra-arm changes, the iso(1q) on left shows two copies of 1q12 and three copies of 1q21, while the iso(1q) on the right shows a large focal amplification of 1q12. The copy number of 1q21 in this cell is five. **e** A cell showing eight copies of 1q12 (arrows) and seven copies of 1q21 distributed among three chromosomes. Left, iso(1q) with three copies 1q12 and two copies of 1q21. Center, iso(1q) with four copies of both 1q12 and 1q21 displaced distally of normal positions. Right single copies of 1q12 and 1q21 on a der(16)

The loss of chromosome 1p by the formation of isochromosome 1q was identified in four patients (nos 4, 6, 7, and 8) with ≥5 of 1q21, with one of these showing complex intra-arm amplification of the 1q12-23 region generated by BFB cycle amplification^19^. Patient no 7 showed the specific progressive expansion of the 1q12-23 region by BFB amplification with 1q12 as the recurring breakpoint in the amplification cycles. In this patient the 1q12-23 amplicon expanded, demonstrating increasing CNAs with four, six, eight, and twelve copies of 1q21 (Fig. 2a–d [top]). In addition to the ladder-like amplification pattern resulting in higher CNAs of 1q12-23, some cells also displayed small extra-chromosomal rings (Fig. 2d [top]). In a second patient (no 4) with iso 1q, extensive cell to cell heterogeneity was found for both copy number and the positioning of 1q12 and 1q21 within the long arm of 1q (Fig. 2a–e [bottom]). These cells showed CNAs for 1q21 which ranged from 4 to 7 and also showed transient features of instability associated with catastrophic chromosome aberrations including localized 1q pulverization and 1q21 micronuclei (Supplemental Fig. 1a–c).

### Deletion of 17p and concomitant gain of 1q21, der(1;17)(q12;q10)

**Fig. 3:**
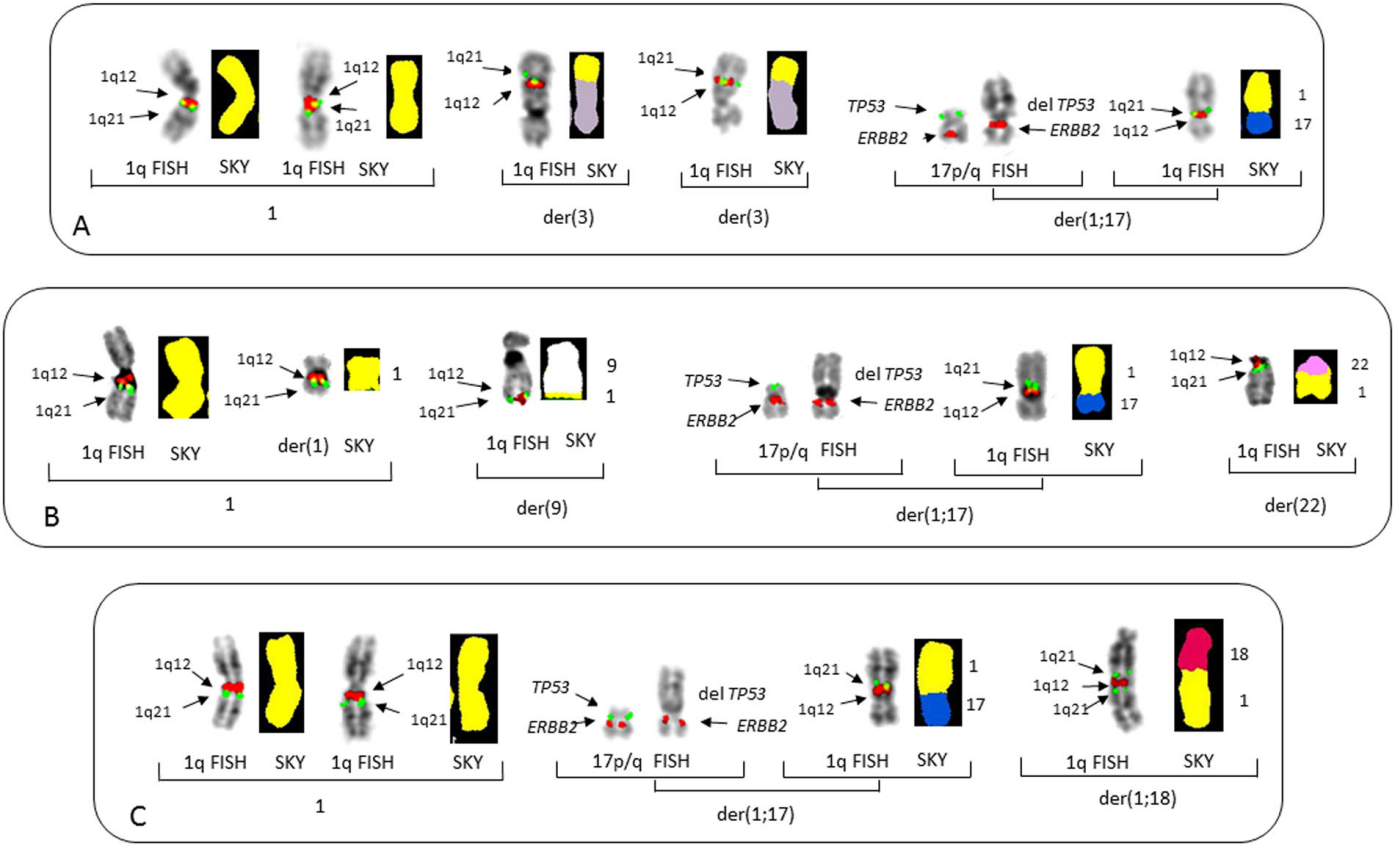
Partial karyotypes of three patients showing concomitant 17p deletions originating from a jumping translocation of 1q12 to 17p. FISH probes for 1q12 (red) and 1q21 (green) are shown on the left. SKY image-classified colors for chromosome 1 (yellow), chromosome 3 (gray), chromosome 9 (white), chromosome 17 (blue), and chromosome 18 (fuchsia), and chromosome 22 (pink). FISH probes for TP53 (green) and ERBB2 (red). **a** Patient no 30 showing chromosomes in brackets from left to right, normal chromosomes 1 with FISH (left) and SKY (right). Middle brackets show two different der(3) RCs both with 1q12 and 1q21. In the brackets on right the chromosomes 17 show hybridization of the normal chromosome 17 with probes TP53 (green) and ERBB2 (red) in their normal positions. The der(1;17) shows the deletion of TP53 (no green signal) and ERBB2 (red) in normal position. In the right bracket the der(1;17) is shown with the 1q replacing 17p and normal positions of 1q12 (red) and 1q21 (green) on 1q. The SKY on the right shows 1q (yellow) replacing 17p and the 17q (blue). **b** Patient no 42 with brackets on far left showing 1q FISH with one normal chromosome 1 (left) and a small der(1) on (right) with large deletions of both 1p and 1q. Bracket middle left shows a der(9) with 1q12 and 1q21 (arrows) on the distal 9q. Brackets middle right shows the normal 17 showing TP53 (green) and ERBB2 (red), the der(1;17) showing loss of TP53 (green) and presence of ERBB2 (red). The bracket on right shows the same der(1;17) chromosome hybridized with 1q12 (red) and 1q21(green) and SKY (right) showing 1q (yellow) replacing the short arm of 17 (blue). Far right bracket shows 1q FISH (left) with 1q12 and 1q21 on 22, and SKY of der(22) with 1q (yellow) translocated to 22 (pink). A total of five copies of 1q21, three of which are distributed to RCs 9q, 17p, and 22q. **c** Patient no 49 in brackets on the left are normal chromosomes 1 by FISH and SKY. Middle brackets show normal TP53 (green) and ERBB2 (red) left and der(17) with deleted 17p (green) by FISH. The brackets on right show normal 1q12 and 1q21 by FISH and 1q replacing 17p (blue) by 1q (yellow) by SKY. Bracket on far right shows an inverted duplication of 1q21 on chromosome 18 with copies of 1q21 on both sides of 1q12 (left) by FISH. SKY showing 1q (yellow) on distal end of 18 (fuchsia)

Deletion of 17p is the single most important iFISH indicator of poor prognosis^5,32,33^ and has previously been found to occur by the JT1q12^20^. In this study five patients showed del(17p) resulting from JT1q12s (nos 3, 30, 42, 43, and 49). Two of these patients had four copies of 1q21, and three showed five copies (nos 30, 42, and 49), all of which showed additional CNAs of 1q21 involving different RCs (Fig. 3a–c). These cases demonstrate that the der(1;17) is a recurring secondary aberration, which involves both an amplification of 1q21 and concomitant deletion of TP53. The additional copies of 1q21 involving different RCs demonstrate the ongoing subclonal heterogeneity introduced by the 1q12 instability.

### Amplification and insertions of MYC and MET

**Fig. 4:**
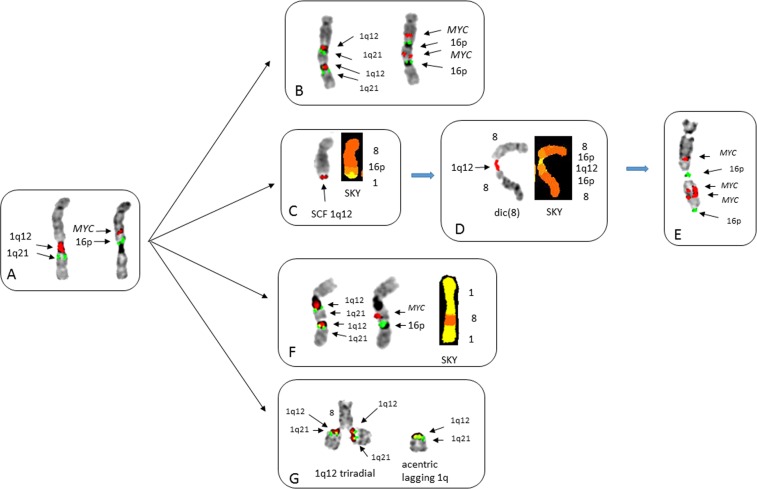
Partial karyotypes of patient no 2 demonstrating the different types of transient 1q12 aberrations that continually regenerate gains of 1q21 and *MYC*. **a** Chromosome 8 showing metaphase FISH of JT1q12 to the der(8) hybridized on left with 1q12 (red) and 1q21 (green) and on right the same chromosome hybridized with MYC (red) and 16p (green). **b** The der(8) (left) hybridized first with chromosome 1 probes demonstrates a direct duplication of 1q12 (red) and 1q21 (green). Importantly, the hybridization with FISH probes for MYC and 16p (on right) shows a cryptic duplication and concomitant amp of the region spanning 1q12-1q21, MYC, and 16p (arrows). **c** Breakage at 1q12 and the loss of 1q distal to 1q12 resulted in a sister chromatid fusion (SCF) at 1q12 on the distal 8q. **d** The SCF aberration resulted in the subsequent formation of a dicentric 8 showing 1q12 (arrow) as the bridging point connecting the dicentric 8. **e** The outcome of BFB cycles of the dicentric (8) is the amplification of MYC and 16p in an inverted duplication pattern typical of BFB amplification. This chromosome has three copies of *MYC* and two of 16p. **f** A dispersed insertion of *MYC* and 16p into one of the previously normal chromosomes 1. **g** The regeneration of additional copies of 1q21 is demonstrated by a triradial of 1q12 (red) on distal der(8q) showing an extra copy of 1q21 (green) transiently attached to each chromatid of the der(8q). Note also in this cell the presence of an extra acentric lagging copy of 1q with signals for both 1q12 (red) and 1q21 (green)

The amplification and distribution of insertions in cancer cells is poorly understood^34^. In this study we found two patients with cryptic unbalanced insertions resulting from 1q12 instability. Patient no 2 showed multiple subclones demonstrating both transient and clonal 1q12 aberrations (Fig. 4). The initial 1q12 aberrations involved a JT1q12 to distal 8q (Fig. 4a). SKY detected a cryptic t(8;16)(q24;p13) had taken place prior to the JT1q12, resulting in the presence of 16p13 inserted between *MYC* and 1q12 on the der(8) (Fig. 4a). Additional progression events included a direct duplication of 1q12-21 on the 8q resulting in extra copies of 1q12, 1q21, *MYC*, and 16p13 (Fig. 4b). BFB cycle amplification (Fig. 4c–e) of both *MYC* and 16p was found, with the initial step being the loss of 1q, followed by a sister chromatid fusion (SCF) at 1q12 (Fig. 4c). The SCF resulted in the formation of an unstable 8q dicentric with 1q12 as the bridging point (Fig. 4d) and subsequent BFB cycle amplification of *MYC* and 16p (Fig. 4e). In a different subclone, a translocation/insertion of both *MYC* and 16p into the 1q12 region of one of the previously normal chromosome 1 homologs was found (Fig. 4f)^18,20^. This insertion could arise following the ongoing instability of 1q12 demonstrated by the presence of the hallmark 1q12 triradial on the distal 8q in the same cell as an extra acentric lagging copy of 1q (Fig. 4g). These transient 1q12 aberrations demonstrate first, that the continuous regeneration of 1q12 triradials occurs on nonhomologous RCs, and secondly this mechanism can cause dispersed insertions of nonhomologous chromosome segments, such as MYC translocations. Additional transient aberrations in this patient included 1q12 micronuclei, interphase bridging of 1q12, and acentric iso(1q)s (Supplemental Fig. 2).

**Fig. 5:**
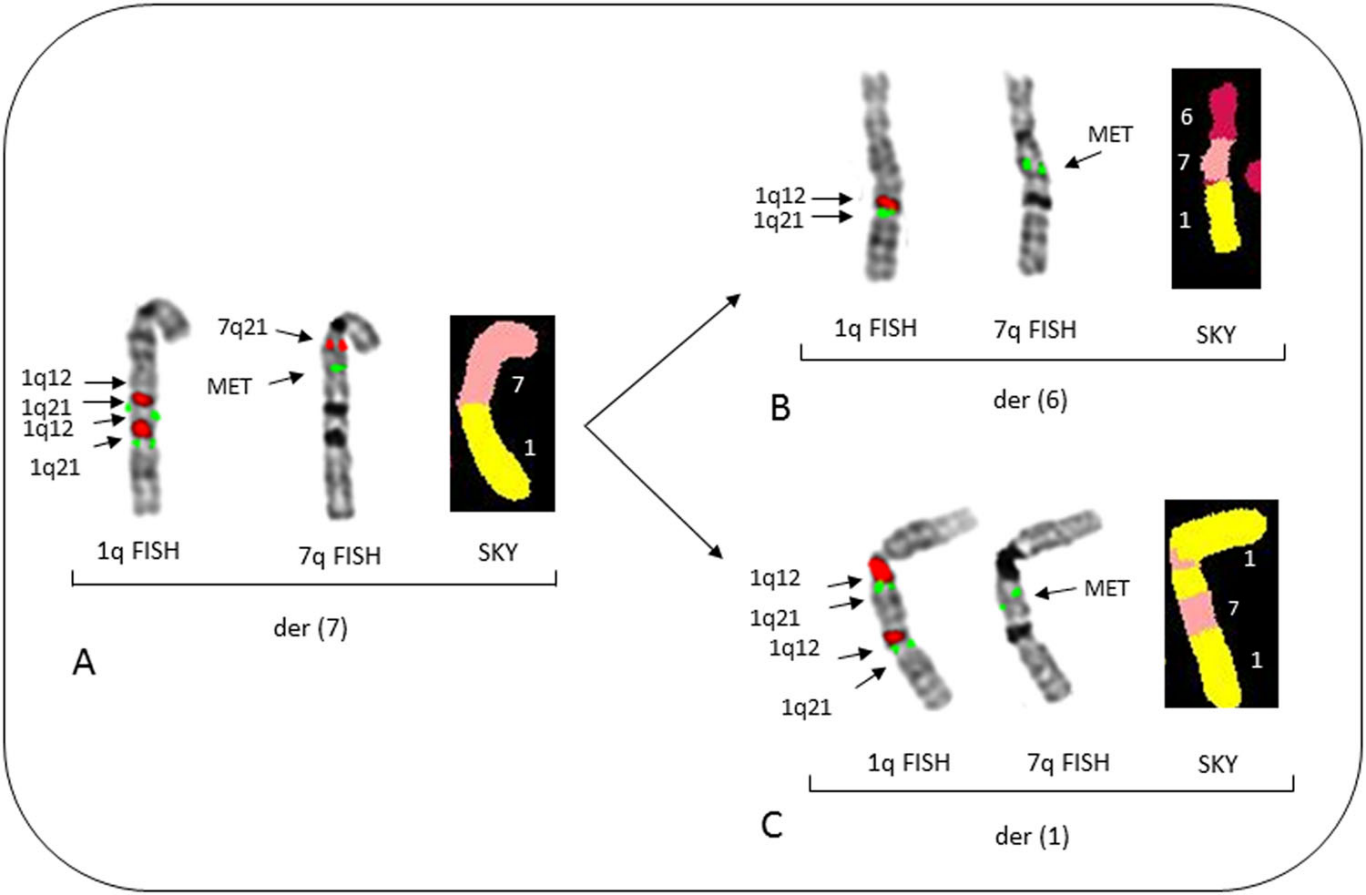
A partial karyotype of patient no 14 showing multiple JT1q12s resulting in dispersed insertions of the MET region (7q31). This clone shows a total of six copies of 1q12 and 1q21, and four copies of MET are present in this cell, normal chromosomes 1, 6, and 7 not shown. **a** Metaphase FISH shows two copies each for 1q12 (red) and 1q21(green) on the der(7q) (left), rehybridization with FISH probes for 7q21 (red) and MET (7q31, green) (middle) indicates the normal locations for these loci, and SKY classification colors (right) shows 1q (yellow) on distal 7q (salmon). **b** FISH probes for 1q12 (red) and 1q21 (green) shows one copy of both probes translocated on 6q (left), when rehybridized with MET (7q31, green), the 6q shows MET is inserted resulting in a der(6) with extra copies of 1q12, 1q21, and MET (middle). The SKY on right shows 6 (magenta), 7q MET regions (salmon) in middle, and 1q (yellow) distal. **c** Fish probes for 1q12 and 1q21 (left) shows an apparent direct duplication on 1q; the same chromosome 1 rehybridized with MET (middle) shows an insertion of MET into the 1q12 pericentromeric region of chromosome 1. On right SKY colors show insertion of 7q (salmon) material into chromosome 1q (yellow)

The amplification and dispersed insertion of the 7q31region containing the MET loci was identified in patient no 14. This patient demonstrated the amplification of both the MET region and 1q21 to 6q and 1q (Fig. 5). In this patient a JT1q12 to 7q occurred where a direct duplication of 1q12-23 occurred (Fig. 5a). Subsequently, a JT1q12 to 6q carrying the MET region caused a deletion in the 6q (Fig. 5b). A second JT1q12 resulted in an insertion of MET into the 1q12 region of one of the previously normal chromosomes 1 (Fig. 5c). Of note, patient no 14 also showed nuclear budding, interphase bridging of 1q12, and multiple variations of acentric lagging 1qs leading to cells with 1q21 counts of 7–8 (Supplemental Fig. 3). Both patients, nos 2 and 14, add to the previous reports demonstrating insertions of *MYC*, BCL2, 16q11, and 20q11 into the 1q12 region, are recurring aberrations of this phenotype^18,20^.

### Major and minor metaphase aberrations identifying the 1q12 instability phenotype

**Fig. 6:**
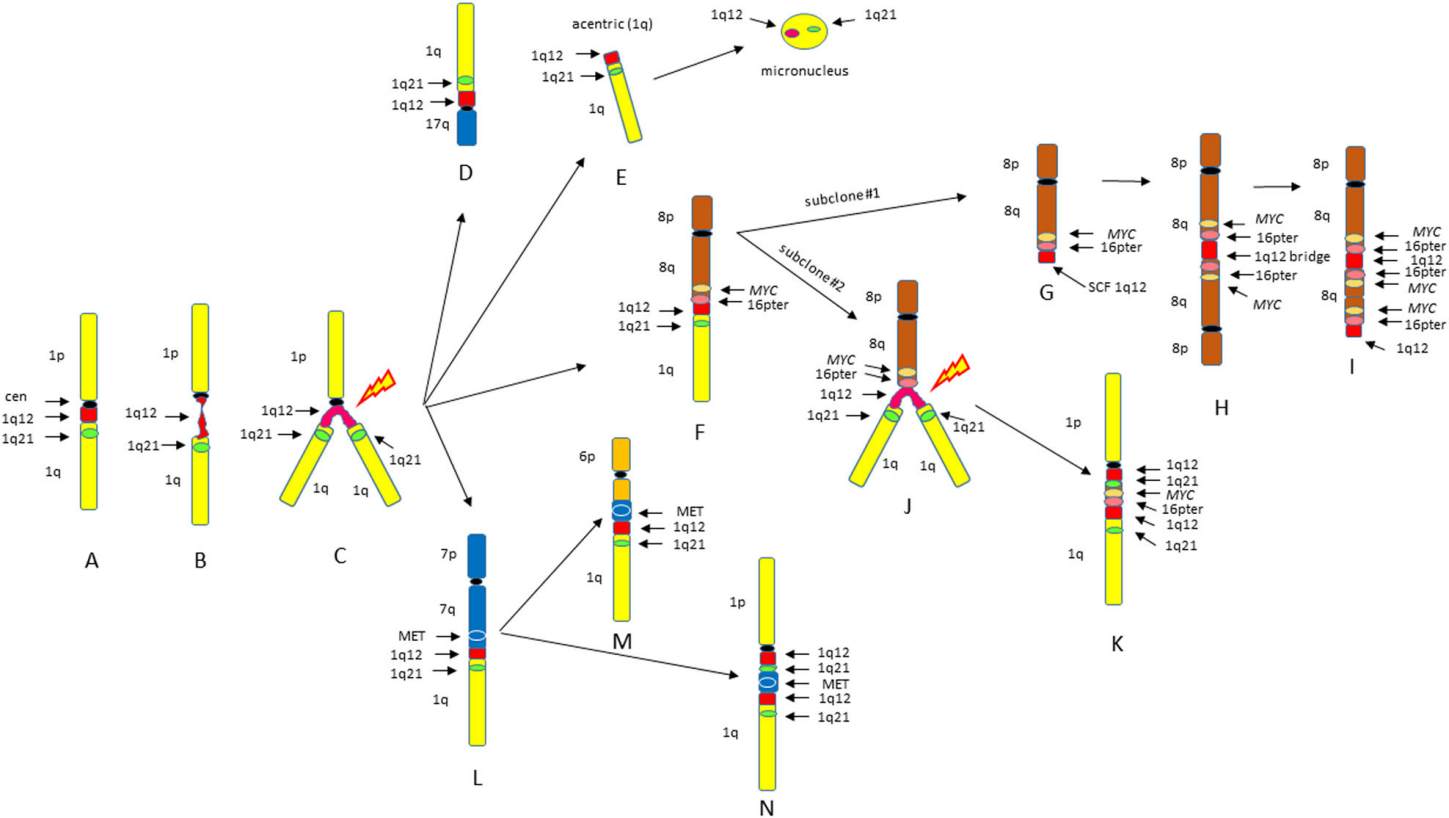
Characterization of possible types of JT1q12 chromosome instability. Depiction of normal chromosome 1 (yellow), centromere (black), 1q12 (red), and 1q21 (green) (**a**). Two transient aberrations of 1q12 are precursors to subsequent clonal aberrations, the first being 1q12 decondensation and breakage (**b**), which leads to the formation of 1q12 triradials (**c**). The extra copy of 1q generated by the triradial of 1q12 can result in multiple types of unbalanced CNAs. A JT1q12 to 17p can result in the concomitant gain of both 1q12 and 1q21 and deletion of 17p (TP53) (**d**). Extra copies of 1q12 that do not successfully jump to an RC result in acentric lagging copies of 1q and subsequent micronuclei (**e**). A JT1q12 to 8q (**f**) demonstrates ongoing instability including the two sublcones. Subclone 1 involved the loss of 1q from 8q, resulting in a sister chromatid fusion (SCF) (arrow) at 1q12 (**g**). Subsequent formation of dicentric 8 and a 1q12 bridge (**h**) is an intermediate step in BFB cycles resulting in the amp of *MYC* and 16pter (**i**). In subclone 2, 1q12 instability is demonstrated by a triradial of 1q on 8q resulting in the generation of additional copies of 1q21 (**j**). The additional copies of *MYC*, 16p, 1q12, and 1q21 are subsequently translocated to a normal chromosome 1q resulting in an insertion (**k**). A JT1q12 to 7q (**l**) subsequently results in insertion of MET into multiple nonhomologous chromosomes, including both 6q, causing a near arm-length deletion in 6q (**m**), and also to an RC 1q resulting in insertion of MET into a normal chromosome 1 (**n**)

From this group of eight patients with ≥5 1q21s (nos 2, 4, 7, 14, 22, 30, 42, 49), and 21 patients from previous reports^18–21^, we identify four major recurring types of clonal cytogenetic aberrations that can be used to define this 1q12 site-specific instability phenotype. These include: (1) JT1q12s to an RC, (2) arm-length deletions in RC, (3) BFB cycle amplifications, and (4) 1q12 insertions. In addition, five types of recurring transient aberrations that define this phenotype include: (1) 1q12 decondensation and breakage, (2) 1q12 triradials, (3) acentric lagging 1q12, (4) 1q12 micronuclei, and (5) localized pulverization of 1q. The key structural chromosome rearrangements defining this CIN phenotype are depicted in Fig. 6. In normal cells, the 1q12 pericentromeric region remains condensed in metaphase (Fig. 6a). Cells showing the transient decondensation and breakage of 1q12 (Fig. 6b) are precursors to triradial formation at 1q12 as seen in patient nos 2 and 22 (Fig. 6c). The extra copy of 1q generated by the triradial results in a JT1q12 and can become a clonal arm-length duplication/deletion as depicted for loss of 17p (Fig. 6d). Alternatively, breakage at 1q12 can result in an acentric copy of 1q12 (Fig. 6e) which subsequently is lost as a micronucleus. JT1q12s to telomeres (Fig. 6f) can remain stable or can further evolve to different sublcones. For example, in patient no 2 the der(8) in subclone 1 (Fig. 6g) evolved by breakage at 1q12 and the loss of 1q, which initiated the formation of a SCF at 1q12. This SCF resulted in the formation of a dicentric der(8) (Fig. 6h), which in turn resulted in BFB cycles amplifying MYC and 16p (Fig. 6i). In subclone no 2, a triradial on the der(8) (Fig. 6j), is depicted with the subsequent 1q12 insertion of MYC and 16p into the 1q (Fig. 6k). In patient no 14 unbalanced dispersed insertions of the MET region of chromosome 7q31 (Fig. 6l) into different RCs result in a duplication of MET and deletion involving 6q (Fig. 6m), and an insertion of MET into 1q12 (Fig. 6n).

### iFISH Identification of 1q12 Instability Phenotype

The jumping 1q12 instability phenotype is identified by iFISH by the presence of ≥5 CNAs of 1q21 and at least one other high-risk iFISH lesion, including high-risk IGH translocations and del(17p). In addition, deletions of 1p detected by iFISH can indicate either an interstitial deletion of 1p or iso(1q), both of which are associated with 1q12 instability.

## Discussion

Chromosomal instability contributes to malignant transformation by altering gene dosage and is known to play a role in tumor progression, relapse, and therapeutic failures^1,3^. The hallmark feature of CIN is the continuous regeneration of either whole-chromosome or segmental CNAs, the causes of which include mitotic segregation errors, aberrant DNA repair, DNA replication stress, and fragile sites, among others^3,4^. Although these mechanisms can account for many of the numerical and structural aberrations found in MM, accumulating evidence indicates that additional mechanisms, such as epigenetic modifications and transcriptome remodeling, likely play a role in the spectrum of segmental CNAs found in relapsed and refractory patients. The presence of 1q12 DNA breaks, localized chromosome pulverization distal to 1q12, acentric lagging chromosomes 1q, and 1q12 micronuclei as described here are part of a spectrum of catastrophic chromosome aberrations associated with chromothripsis^35^. In fact, localized pulverization of chromosome arms in micronuclei has been suggested as one mechanism for chromothripsis^36^. Interestingly, chromothripsis has been reported in MM involving a rare, aggressive entity in some patients with 1q aberrations^37^.

The CIN phenotype described here is acquired during disease progression or at relapse with the accumulation of a combination of adverse CNAs of 1q21, 17p, and IgH translocations. This cytogenetic phenotype is characterized by the continuous regeneration of site-specific 1q12 aberrations that propagate and induce an apparently unlimited number of both transient and clonal 1q12 aberrations (Figs. 1 and 3). Four recurring 1q12 cytogenetic features of this phenotype include: (1) JT1q12s to the RCs, (2) 1q12 induced deletions in RCs, (3) 1q12 induced BFB cycle amplifications, and (4) 1q12 insertions (Fig. 6). In addition, five transient features include: (1) 1q12 decondensation and breakage, (2) 1q12 triradials, (3) acentric lagging 1q12, (4) 1q12 micronuclei, and (5) localized pulverization of 1q distal to 1q12.

The cause of this cytogenetic phenotype is currently unknown; however, it has been speculated that hypomethylation or other modifications to the 1q12 region are responsible^17^. The 1q12 pericentromeric heterochromatin is made up of the single largest block of late-replicating highly repetitive satellite DNA in the human genome, contains a 1q12 fragile site, and undergoes satellite repeat expansions in cancer^38,39^. In normal cells this region remains highly condensed; however, in tumor cells this region can become perturbed, decondensed and prone to breakage and jumping as occurs in ICF syndrome. ICF patients have a known mutation in DNA methyltransferase (DNMT) 3B, which is thought to be responsible for the site-specific hypomethylation of the 1q12 pericentromeric heterochromatin and 1q12 instability^40^. In MM and other B-cell disorders no DNMT mutations known to affect 1q12 have been reported, although global DNA hypomethylation of repetitive elements and aberrant methylation patterns have been reported as associated with disease progression^41^.

Accumulating evidence also now suggests modifications of the 1q12 region by other genetic and epigenetic factors. For example, reprogramming of 1q12 pericentromeric satellite DNA domain has been reported to be associated with polycomb body formation on the 1q12 satellite DNA. This epigenetic conversion coincides with both global and satellite DNA demethylation, and has been shown to be induced by inhibition of DNMTs^42^. Another possible cause of instability is the overexpression of the chromatin modifying enzymes, such as KDM4A. KDM4A is a histone demethylase, which binds the BCL9 locus and causes the rereplication and site-specific copy number gains of 1q12 and 1q21^43^, altered expression of microRNAs, and selective pressures, such as hypoxic stress^44–46^. The induced gains of 1q12 and 1q21 reported in these studies are not stably inherited but transiently regenerated in subsequent S phases, just as in ICF syndrome and MM. The in vitro modification of the 1q12 region by 5-azacytidine also provides evidence that the site-specific hypomethylation of the 1q12 region can induce copy number gains of 1q21 and regions translocated next to it^47^. This finding demonstrates that 1q12 pericentromeric heterochromatin can potentially drive CNAs of any genomic region juxtaposed to it.

Alterations in RNA processing have also been linked to 1q21 CNAs through amplification and overexpression of ILF2. ILF2 is a gene, which promotes tolerance of genomic instability and the stabilization of transcripts involved in homologous recombination (HR). The upregulation of 1q21 by ILF2 deregulates HR resulting in increased abnormal nuclear morphologies including nucleoplasmic bridges, nuclear buds, and micronuclei^23^, demonstrating the same types of chromosomal instability characterized here (Supplemental Figs. 1–3). Elevated levels of HR are thought to mediate genomic instability, an increase in mutations, and the accumulation of genetic alterations in MM^48^. Finally, RNA editing in MM of ADAR1, an adenosine deaminase acting on RNA-1 in the 1q21 region, has been reported to promote malignant regeneration resulting in reduced survival in MM^49^.

Recent genomic studies have shown that most patients already have complex subclonal structure at diagnosis that undergoes further evolution over time^26,27^. The chronology of CNAs in MM suggests a greater degree of instability is associated with complex deletions and a punctuated evolutionary process^50^. This is in agreement with our findings that patients with more advanced disease show the onset of chromosome 1q12 instability and the accumulation of ≥5 copies of 1q21 in association with the deletions of 17p and other regions^17–21^. Here we define a high-risk cytogenetic phenotypic as a constellation of cytogenetic findings identifiable by current iFISH panels. In this subset of patients, the 1q12 satellite DNA acquires a self-propagating mobile property that adversely impacts the genome by initiating whole-arm JT1q12 and concomitant deletions in RCs. As part of this phenotype, additional smaller focal duplications/triplications of 1q21 and other regions occur, evidenced by BFB cycles and insertions. We propose the term “Jumping 1q Syndrome” for this cytogenetic phenotype type based on the two factors. First, the striking mechanistic relationships of the 1q12 aberrations shared with ICF syndrome and second on the four characteristic patterns of aberrations associated with induced by 1q12 instability. CIN phenotypes enable cells to enter different evolutionary trajectories and adapt to selective pressures of therapies, which underlies treatment failures^4^. Given the low prevalence of mutated driver genes in MM, and the stronger impact of cytogenetic aberrations on prognosis over gene mutations^51^, it may be that the apparently unlimited combination of unbalanced 1q12 amplifications/deletions is in part responsible. This study suggests that these patients may exhibit a previously unrecognized form of ultra-high-risk disease^52,53^, and provides evidence that patients with ≥5 CNAs of 1q21 have a type of emerging epigenetic instability in need of novel types of therapeutic intervention^54–56^.

## Supplementary information

Supplematal Table and Figure Legends

Supplemental Figure 1

Supplemental Figure 2

Supplemental Figure 3

Supplemental Table 1

Supplemental Table 2

## References

[1] Lengauer C, Kinzler KW, Vogelstein B (1998). Genetic instabilities in human cancers. Nature..

[2] Geigl JB, Obenauf AC, Schwarzbraun T, Speicher MR (2008). Defining chromosomal instability. Trends Genet..

[3] Bakhoum SF, Landau DA (2017). Chromosomal instability as a driver of tumor heterogeneity and evolution. Cold Spring Harb. Perspect. Med..

[4] Sansregret L, Vanhaesebroeck B, Swanton C (2018). Determinants and clinical implications of chromosomal instability in cancer. Nat. Rev. Clin. Onclol..

[5] Kumar SK, Rajkumar SV (2018). The multiple myelomas—current concepts in cytogenetic classification and therapy. Nat. Rev. Clin. Oncol..

[6] Manier S (2017). Genomic complexity of multiple myeloma and its clinical implications. Nat. Rev. Clin. Oncol..

[7] Chng WJ, International Myeloma Working Group. (2014). IMWG consensus on risk stratification in multiple myeloma. Leukemia.

[8] Sonneveld P (2016). Treatment of multiple myeloma with high-risk cytogenetics: a consensus of the International Myeloma Working Group. Blood.

[9] Boyd KD, NCRI Haematology Oncology Studies Group. (2012). A novel prognostic model in myeloma based on co-segregating adverse FISH lesions and the ISS: analysis of patients treated in the MRC myeloma IX trial. Leukemia.

[10] Walker BA (2006). Integration of global SNP-based mapping and expression arrays reveals key regions, mechanisms, and genes important in the pathogenesis of multiple myeloma. Blood.

[11] Avet-Loiseau H (2009). Prognostic significance of copy-number alterations in multiple myeloma. J. Clin. Oncol..

[12] Walker BA (2010). A compendium of myeloma-associated chromosomal copy number abnormalities and their prognostic value. Blood.

[13] Avet-Loiseau H (2012). Long-term analysis of the IFM 99 trials for myeloma: cytogenetic abnormalities [t(4;14), del(17p), 1q gains] play a major role in defining long-term survival. J. Clin. Oncol..

[14] L´opez-Corral L (2012). SNP-based mapping arrays reveal high genomic complexity in monoclonal gammopathies, from MGUS to myeloma status. Leukemia.

[15] Beroukhim R (2010). The landscape of somatic copy-number alterations across human cancers. Nature.

[16] Davoli T (2013). Cumulative haploinsufficiency and triplosensitivity drive aneuploidy patterns and shape the cancer genome. Cell.

[17] Sawyer JR, Tricot G, Mattox S, Jagannath S, Barlogie B (1998). Jumping translocations of chromosome 1q in multiple myeloma: evidence for a mechanism involving decondensation of pericentromeric heterochromatin. Blood.

[18] Sawyer JR (2005). Genomic instability in multiple myeloma: evidence for jumping segmental duplications of chromosome arm 1q. Genes Chromosom. Cancer.

[19] Sawyer JR (2009). Evidence for a novel mechanism for gene amplification in multiple myeloma: 1q12 pericentromeric heterochromatin mediates breakage-fusion-bridge cycles of a 1q12-23 amplicon. Br. J. Haematol..

[20] Sawyer JR (2014). Jumping translocations of 1q12 in multiple myeloma: a novel mechanism for deletion of 17p in cytogenetically defined high-risk disease. Blood.

[21] Sawyer JR (2017). Hyperhaploidy is a novel high-risk cytogenetic subgroup in multiple myeloma. Leukemia.

[22] Shaughnessy JD (2007). A validated gene expression model of high-risk multiple myeloma is defined by deregulated expression of genes mapping to chromosome 1. Blood.

[23] Marchesini M (2017). ILF2 is a regulator of RNA splicing and DNA damage response in 1q21-amplified multiple myeloma. Cancer Cell..

[24] Pawlyn C, Morgan GJ (2017). Evolutionary biology of high-risk multiple myeloma. Nat. Rev. Cancer.

[25] Hanamura I (2006). Frequent gain of chromosome band 1q21 in plasma-cell dyscrasias detected by fluorescence in situ hybridization: incidence increases from MGUS to relapsed myeloma and is related to prognosis and disease progression following tandem stem-cell transplantation. Blood.

[26] Walker BA (2018). Identification of novel mutational drivers reveals oncogene dependencies in multiple myeloma. Blood.

[27] Walker BA (2018). A high-risk, double-hit, group of newly diagnosed myeloma identified by genomic analysis. Leukemia.

[28] Weemaes CM (2013). Heterogeneous clinical presentation in ICF syndrome: correlation with underlying gene defects. Eur. J. Hum. Genet..

[29] Gardner R.J.M, Sutherland Grant R, Shaffer Lisa G. (2011). Chromosome Instability Syndromes. Chromosome Abnormalities and Genetic Counseling.

[30] Sawyer JR, Swanson CM, Wheeler G, Cunniff C (1995). Chromosome instability in ICF syndrome: formation of micronuclei from multibranched chromosomes 1 demonstrated by fluorescence in situ hybridization. Am. J. Med. Genet..

[31] International system for human cytogenetic nomenclature. in: McGovan-Jordan J., ed An International System for Human Cytogenetic Nomenclature. (Basel S. Karger, Unionville, CT, 2016).

[32] Shah V (2018). Subclonal *TP53* copy number is associated with prognosis in multiple myeloma. Blood.

[33] Thakurta Anjan, Ortiz Maria, Blecua Pedro, Towfic Fadi, Corre Jill, Serbina Natalya V., Flynt Erin, Yu Zhinuan, Yang Zhihong, Palumbo Antonio, Dimopoulos Meletios A., Gutierrez Norma C., Goldschmidt Hartmut, Sonneveld Pieter, Avet-Loiseau Herve (2019). High subclonal fraction of 17p deletion is associated with poor prognosis in multiple myeloma. Blood.

[34] Albertson DG (2006). Gene amplification in cancer. Trends Genet..

[35] Ly P, Cleveland DW (2017). Rebuilding chromosomes after catastrophe: emerging mechanisms of chromothripsis. Trends Cell Biol..

[36] Crasta K (2012). DNA breaks and chromosome pulverization from errors in mitosis. Nature.

[37] Magrangeas F, Avet-Loiseau H, Munshi NC, Minvielle S (2011). Chromothripsis identifies a rare and aggressive entity among newly diagnosed multiple myeloma patients. Blood.

[38] Fournier A (2007). Genetics and epigenetics of 1q rearrangements in hematological malignancies. Cytogenet Genome Res..

[39] Bersani F (2015). Pericentromeric satellite repeat expansions through RNA-derived DNA intermediates in cancer. Proc. Natl Acad. Sci. USA.

[40] Xu GL (1999). Chromosome instability and immunodeficiency syndrome caused by mutations in a DNA methyltransferase gene. Nature.

[41] Bollati V (2009). Differential repetitive DNA methylation in multiple myeloma molecular subgroups. Carcinogenesis..

[42] Brückmann NH, Pedersen CB, Ditzel HJ, Gjerstorff MF (2018). Epigenetic reprogramming of pericentromeric satellite DNA in premalignant and malignant Lesions. Mol. Cancer Res..

[43] Black JC (2013). KDM4A lysine demethylase induces site-specific copy gain and rereplication of regions amplified in tumors. Cell.

[44] Black JC (2015). Hypoxia drives transient site-specific copy gain and drug-resistant gene expression. Genes Dev..

[45] Black JC, Zhang H, Kim J, Getz G, Whetstine JR (2016). Regulation of transient site-specific copy gain by microRNA. J. Biol. Chem..

[46] Wu C (2018). ARNT/HIF-1β links high-risk 1q21 gain and microenvironmental hypoxia to drug resistance and poor prognosis in multiple myeloma. Cancer Med..

[47] Sawyer JR (2015). Evidence of an epigenetic origin for high-risk 1q21 copy number aberrations in multiple myeloma. Blood.

[48] Shammas MA (2009). Dysfunctional homologous recombination mediates genomic instability and progression in myeloma. Blood.

[49] Lazzari E (2017). Alu-dependent RNA editing of GLI1 promotes malignant regeneration in multiple myeloma. Nat. Commun..

[50] Aktas Samur A (2019). Deciphering the chronology of copy number alterations in multiple myeloma. Blood Cancer J..

[51] Bolli N (2018). Analysis of the genomic landscape of multiple myeloma highlights novel prognostic markers and disease subgroups. Leukemia.

[52] Avet-Loiseau H (2010). Ultra high-risk myeloma. Hematol. Am. Soc. Hematol. Educ. Prog..

[53] Usmani SZ, Rodriguez-Otero P, Bhutani M, Mateous MV, Miguel JS (2015). Defining and treating high-risk multiple myeloma. Leukemia.

[54] Jones PA, Ohtani H, Chakravarthy A, De Carvalho DD (2019). Epigenetic therapy in immune-oncology. Nat. Rev. Cancer.

[55] Harding T, Baughn L, Kumar S, van Ness B (2019). The future of myeloma precision medicine: integrating the compendium of known drug resistance mechanisms with emerging tumor profiling technologies. Leukemia.

[56] Alzrigat M, Párraga AA, Jernberg-Wiklund H (2018). Epigenetics in multiple myeloma: from mechanisms to therapy. Semin. Cancer Biol..

