# Supplementary material for: An acquired high-risk chromosome instability phenotype in multiple myeloma: Jumping 1q Syndrome
Source: Blood Cancer J. 2019 Aug 9;9(8):62. doi: 10.1038/s41408-019-0226-4 (PMC6689064; doi:10.1038/s41408-019-0226-4)
Supplement: Supplementary file 1 — Supplematal Table and Figure Legends [file 41408_2019_226_MOESM1_ESM.docx]

This file contains Supplemental Table and Figure legends.

**Supplemental Table 1.** **Clinical features and cytogenetic aberrations of 50 patients selected based CNAs of > 4 copies for 1q21.** Clinical features including age, sex, IgH iso type, L-chain, ISS stage, and cytogenetic features including ploidy, IgH translocations, copy number of 1q12 and 1q21, and copy number of 17p. Chromosome ploidy levels defined by hyperdiploid (HRD) equaling 47 to 75 chromosomes and non-hyperdiploid (NHRD) equaling 46 and/or >75 chromosomes. Receptor chromosome (RC) 1q translocations and deletions.

**Supplemental Table 2.** **Complete G-band karyotype findings for all fifty patients.** Complete G-band results for all 50 patients according to the International System for Human Cytogenetic Nomenclature, 2016.

**Supplemental Figure 1**

**Interphase nuclei and metaphase spread from patient # 4 showing presence of both small and larger micronuclei and pulverized 1q.** (A) Micronuclei with a single copy of 1q12 and 1q21 (B) Micronuclei with one copy of 1q12 and two copies of 1q21. (C) Inverted Dapi G-band image of metaphase cell showing pulverized chromosome 1q in side box (left). SKY image of the same metaphase showing fragments of 1q inside box (middle). On right, the karyotype of the metaphase cell showing pulverized 1q inside box.

**Supplemental Figure 2**

**Cells from patient # 2 showing micronuclei, chromatin bridging of interphase cells, and acentric lagging pseudo iso(1q).** (A) Inverted Dapi image showing two copies of 1q12 and three copies of 1q21 in a micronuclei, (B) Inverted Dapi image on left of multiple nuclei showing 1q12 (arrows) bridging between the nuclei, and Dapi image on right. (C) Inverted Dapi image of metaphase spread showing acentric lagging pseudo iso(1q) on left, and Dapi image on right.

**Supplemental Figure 3**

**Interphase and metaphase cells from patient # 14 showing micronucleus budding, interphase bridging and cell to cell variability of lagging 1qs**. These aberrations demonstrate ongoing instability of 1q12 resulting in cell to cell variability with higher copy numbers of 1q21. FISH probes for 1q12(red) and 1q21 (green) (small arrows). (A) Nuclear budding of 1q12 and 1q21 from an interphase nucleus is the precursor to subsequent micronuclei formation, note 1q12 and 1q21 in the budding segment. (B) Bridging of 1q12 heterochromatin between interphase nuclei (arrows). (C) Metaphase cell showing seven copies of 1q21 with a single copy of 1q21 on an acentric lagging 1q (blue arrow). (D) Metaphase cell also showing seven copies of 1q12 and 1q21 with two copies of the probes on an acentric lagging iso(1q) (blue arrow). (E) Metaphase cell showing eight copies of 1q12 and 1q21 with an additional acentric lagging 1q showing two copies of the probes (blue arrow).
