# Supplementary figures and images for: An acquired high-risk chromosome instability phenotype in multiple myeloma: Jumping 1q Syndrome
Source: Blood Cancer J. 2019 Aug 9;9(8):62. doi: 10.1038/s41408-019-0226-4 (PMC6689064; doi:10.1038/s41408-019-0226-4)

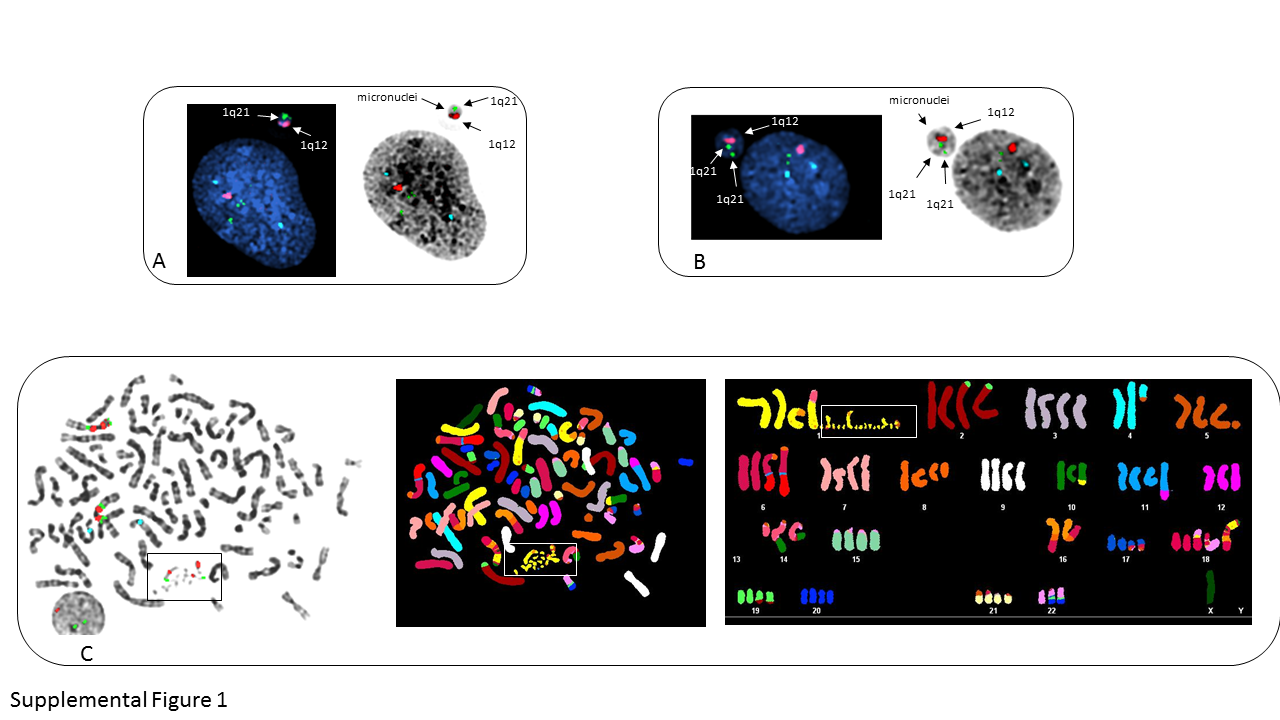

Supplement: Supplementary file 2 — Supplemental Figure 1 [file 41408_2019_226_MOESM2_ESM.tif]

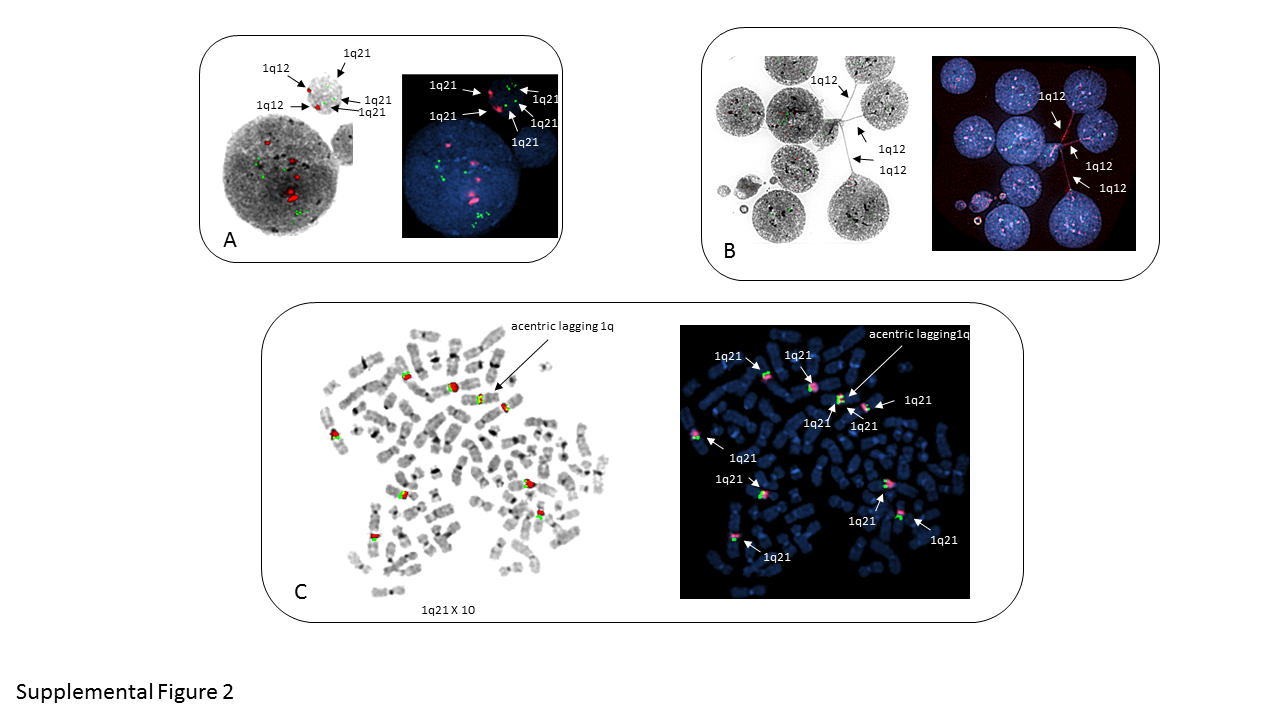

Supplement: Supplementary file 3 — Supplemental Figure 2 [file 41408_2019_226_MOESM3_ESM.tif]

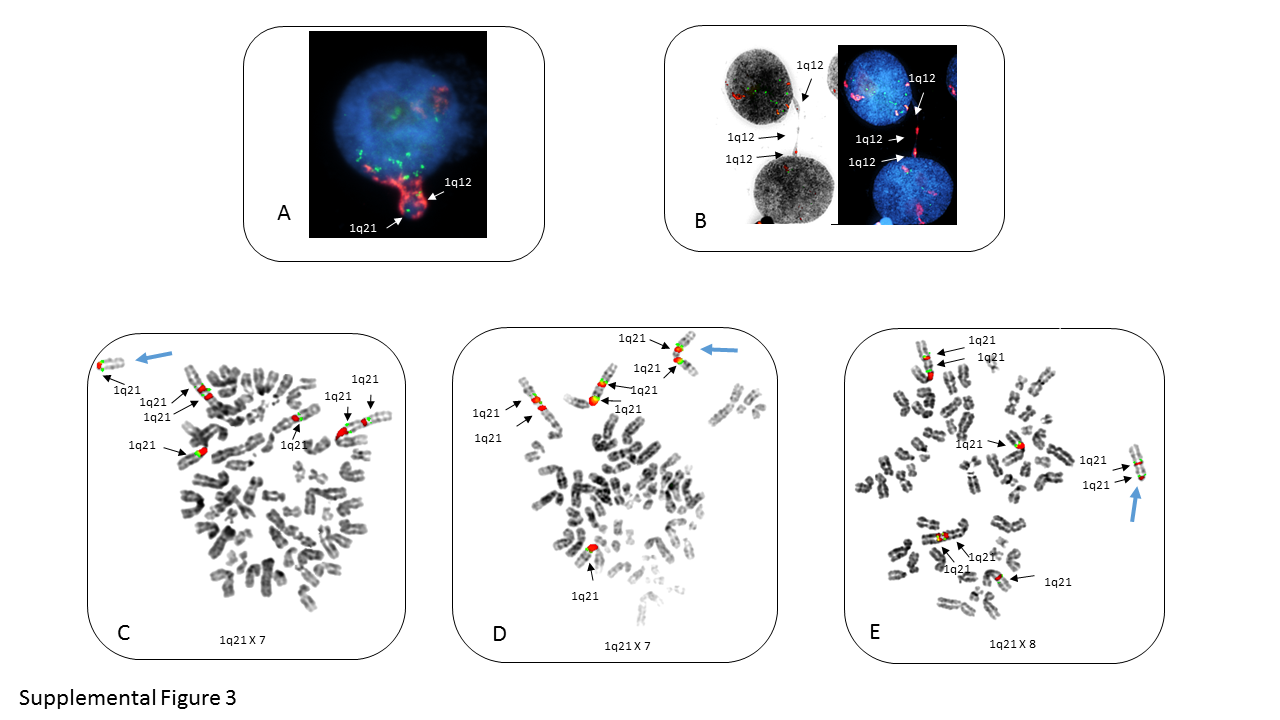

Supplement: Supplementary file 4 — Supplemental Figure 3 [file 41408_2019_226_MOESM4_ESM.tif]
